# Quarantine on the Danube

**Published:** 1838

**Authors:** 


### Art. IV.—A Quarantine on the Danube.

The ridiculous mummery of a Quarantine on the Danube, is so well hit off by Miss Pardoe, in her late work, entitled the "*City of the Sultan*," that we are tempted to transcribe it, for such of our readers as may not have purchased her volumes. When she left Constantinople, the plague was prevailing in that city.

The town and fortress of Orsova occupy an island of considerable length, and have a very picturesque appearance; the gleaming minaret of the solitary mosque cutting against the party-coloured foliage that clothes the hills by which it is overshadowed; and the castellated and buttressed wall of the town reflecting itself in the river-tide. Much of this wall is now in ruin, although it may still be traced entirely along the bank. The island was fortified by the Austrians, but was afterwards ceded to the Turks, together with the fortress of Belgrade by the Emperor Leopold.

From this point we could distinguish the Quarantine establishment, niched in at the foot of the Banut mountains, and distant from the town of Alt Orsova about a mile. But we were obliged to overshoot it by nearly half a league, from the fact of their being no boats for hire until we reached the village of Tekia, situated by the river side, whence the embarkations of the "condemned" universally take place.

When the large flat-bottomed barge in which we were to be conveyed thither, was freighted with our packages, and that we were about to push off, we were detained for an instant by the declaration of the little Servian beauty that she had determined to be of the party; and on board she accordingly came, having flung over her house-costume a magnificent pelisse of gray cloth, eged with sable, and worked with gold.

In half an hour we reached a long wooden shed, built as a receiving house for the quarantine; and here we were detained until our patience was fairly outworn, and that our hunger had become most painful. A double partition of wood parted us from the authorities, who graciously welcomed us to the horrors of incarceration; and we were obliged to seat ourselves on the luggage, and await the arrival of the bullock-carriages that were to convey our travelling-gear to its destination.

All was at last accomplished; and after taking leave of our pretty Servian companion, who laughed heartily at my pressing invitation to her to share our imprisonment; we followed the train of wagons; the rear of the party being brought up by an Austrian soldier, armed with a loaded musket, and a fixed bayonet. We were, however, in no mood to yield to gloomy ideas or feelings. We had a blue sky above us, a fine turf beneath our feet, and the prospect of another half hour of comparative liberty; and we were straggling gaily about the plain, laughing and speculating on our approaching imprisonment, when we were called to order by the guard; and compelled to keep to the high road, lest we should contaminate the grass and thistles among which we were wandering.

Before we reached the quarantine ground, we passed the grave yard destined to receive those who die of plague during their incarceration. It was closely fenced; and rendered still more gloomy by a tall crucifix, painted red, and supporting a most revolting effigy of Our Lord.

On ringing a bell the great gates of the establishment were flung "hospitably" back, and we were requested to allow the wagons to enter before us, lest we should contaminate the oxen by our contact; and after passing through a couple of walled yards, surrounded by warehouses for receiving merchandize, we entered a third enclosure wherein we were met by the governor and surgeon; who keeping at a respectful distance, invited us to enter a dark, white-washed, iron-grated cell, in order to have our passports examined.

A wooden lattice separated us from our new hosts; and the peasant who had conducted us from the river side, stood in front of a small opening made for the purpose, and held at arm's length the papers which were demanded. Much bowing and scraping ensued between M. le Directeur, the foreign Nobleman, and the Hungarian Chevalier; and we had reason to congratulate ourselves on their companionship, as it produced a visible increase of courtesy on the part of the local authorities: a courtesy which did not however, exempt us from the "locks, bolts, and bars" of the Lazaretto. As I was only the second lady who had been unfortunate enough to come under his keeping, the Governor very politely resolved to commence his arrangements by providing me with as good a cell as he had then vacant—not that he called the space into which he was about to consign me, a *cachot*—by no means—the word "cell" being somewhat grating, another term has been invented; and the dens of the Lazaretto of Orsova are designated *colleves*, which signifies—nothing.

But before we could take possession of our prison, another gate had yet to be unlocked; which admitted us into a large space enclosed within a high wall, and containing the *elite* of the accommodations.—The cells, like those of a madhouse in Turkey, were built round the four sides of a garden; and each had a small entrance-court, paved with stone. As none of the buildings were capacious enough to contain our whole party, it was at lenght arranged that five of us should take one of them, in which we might make such arrangements as we preferred; and that the three others should be accommodated as near to us as possible. Upon which understanding, M. le Directeur, a plump, good-natured-looking little old man, with a bit of soiled red ribbon displayed in the button-hole of a threadbare grey frock coat, a ruffled shirt, and the funniest of all forage caps, led the way to Cell, or I should rather say *colleve*, No. 2: and when one of his followers had unlocked the yellow and black gate of the court, he bowed ceremoniously to me, as he pointed to two melancholy-looking trees, which had contrived to exist amid the rude paving, and exclaimed with a tone and gesture perfectly dramatic; "*Soyez la bien venue, Madame; voyez les beaux arbres que vous avez!*"

It was extremely fortunate that the day chanced to be one of cloudless sunshine, and that we consequently saw every thing under its most favorable aspect; for there was nothing particularly exhilarating in the interior of the buildings. Windows both barred and grated; walls whitewashed and weather-stained; chairs, tables, and sofa all of wood, which is a "non-conductor," and whitewashed like the walls; were the only objects that met our eyes. But as we were all both tired and hungry, we welcomed even these; and only begged to learn where we must address ourselves, in order to procure some food with as little delay as possible.

This brought us to the second feature of our position; for a window whose shutter was padlocked up, was unfastened; a bell was rung, and at a casement grated like our own appeared the Restaurateur of the Lazaretto to receive his instructions. Dinner was instantly ordered; bread and wine were speedily procured; and we were waited upon by a very gaily-dressed, conceited individual, who announced himself to be "our keeper;" a piece of intelligence which once more carried back my thoughts to the *Timerhazes*, or madhouses of Constantinople; and I began half to apprehend that we had indeed intruded into one of those melancholy establishments. At five o'clock we were furnished with a very bad dinner; bedding was brought in; and at sunset we were locked up.

On the morrow we were somewhat disconcerted to learn that the court of the *colleve* was to be our boundary during the ten days of our imprisonment: and our officious "keeper" very carefully locked the gate every time that he thought proper to make his escape. But this was a trifling annoyance to that by which it was succeeded; and which consisted of an announcement that at midday the Surgeon of the Lazaretto, and the Examining Officer, would visit us, in order to take an inventory of every thing in our possession. Each trunk, portmanteau, and basket, was to be unpacked; in short, we were even to declare the contents of our purses!

We were already aware that the Austrian was the most paternal of all Governments; taking an interest in the private affairs, not only of its own subjects, but also in those of strangers; yet I confess for such a proceeding as the present we were totally unprepared.

There was, however, no remedy; and the "secret recesses" of every package were laid bare before the "authorities." The reason given for this inconvenient and revolting stretch of power, is the desire of the Government that, in the event of a decease, the friends of the dead person may receive every part of his property upon demand; the inventory held by the proper officers effectually preventing the keeper of the *colleve* from plundering the trunks; but certain little circumstances which we remarked during the investigation rather tended to weaken our faith in the disinterestedness of the arrangement.

When the possession of any Turkish article was mentioned, there was a visible excitement. Even a lantern exhibited by my father was entered on the list; and the number of chibouk-tubes, of tobacco purses and other trifles, which could be of no value to the survivors of a deceased person, were registered with equal exactness.

In my own case they were peculiarly inquisitive; counting my rings, and recording my bracelets and necklaces. Not a pocket-handkerchief, nor a waiste-ribbon escaped; and I was more than once asked if I had really exhibited the whole of my wardrobe. My books and drawings were seized without ceremony, and carried off to be examined by the proper officer; and the worthy functionaries at length departed in full possession of all which related to our peripatetic properties.

It required a couple of hours to soften down the "chafed humours" of the gentlemen of the party; which were not rendered more gentle by the demand of the keeper, that they should deliver up all their arms, of whatever description they might be; on the understanding that they were to be restored to them on the day of their own delivery. But the request did not meet with the ready acquiescence which had been anticipated. Colonel ——— had travelled with the whole of his uniform; and when our attendant advanced to lay sacrilegious hands upon his sword, which was hanging over a chair, all the quick sense of honour of the British soldier was roused at once; and, as the indignant blood rushed to his brow, he vowed that he would fell to the earth the first man who dared to meddle with his side-arms. In vain did the keeper insist, and the Chevalier explain; the English heart beat too high to heed either the one or the other: and the pistol-laden functionary was obliged to depart without the sword of the gallant Guardsman. Of course he made his report to the Governor; but the worthy little old gentleman had too much good sense to persist in the demand; and no allusion was afterwards made to the subject.

Twice each day we were visited by the medical officer, who just popped his head in at the door, and smiled forth: "Ah! quite well, quite well, I see—impossible to be better—good morning," away he went, without affording us time to complain had we been so inclined. M. le Directeur also paid us several visits, always carefully pointing his cane before him, as a warning to us not to approach him too closely: and never failing to commence the conversation by the ejaculation of, "*Madame, je vous salue—ha! les beaux arbres que vous avez!*" It was really worse than ludicrous.

As a signal mark of favour, we were occasionally permitted to walk, under the charge of the keeper, from the gate of our own *colleve*-court to that of our friends, and to receive their visits in return, when we had always a very laughable interview; the incarcerated individuals amusing themselves by rocking to and fro behind the bars of their prison-grates, and roaring like wild beasts in a menagerie.

There are two descriptions of persons to whom I would particularly recommend an avoidance of the Quarantine at Orsova. The *ennuye* and the *bon vivant.* For the first there is no refuge save sleep, and the few doggrel attempts at poetry which may be partially traced thro' the white-wash; the outpourings of an impatient spirit weary of its thrall; with the occasional society of the "keeper," who is as cold and as impracticable as his own keys. The very books of which the wanderer has made his travelling companions; and some of which would bear a second perusal, at all events in a quarantine cell, are carried off and sealed up, as though every volume were redolent of high treason; and he is left to his own resources as ruthlessly as if he were indeed "the last man;" and that he had done with the world and the world with him.

To the second I need only hint that the *restaurant* is a Government monoply, where you are provided for at a fixed sum per day; and fed upon whatever it may please the Comptroller of the Kitchen to serve up. Nor can you procure any wine save the sour and unpalatable *vin du pays*, however liberally you may be disposed to pay for it.

Those travellers are fortunate, who, like ourselves, can meet the captivity of quarantine with pleasant companions, light hearts, and unfailing spirits; finding food for mirth in their very miseries; and forgetting the annoyance of present detention in the anticipation of future freedom.

The last day of our captivity was the most tedious portion of the whole, for the prospect of speedy emancipation kept us in a constant state of irritation. Our luggage was collected and arranged with a haste which by no means added to its comfort or convenience, and which only left us an additional hour of unoccupied restlessness; while the servants were urged to a continual commotion that robbed us even of the tranquility which might have made our prison-house somewhat more endurable.

The morning of the fifteenth of October was that of our release.—We were all ready to depart at daybreak; and after the necessary ceremonies had been gone through, we assembled in a large grassy space, bounded on one side by the Danube, and skirted on the other by the Quarantine buildings. This enclosure was crowded with oxen, wagons, and bales of merchandize; and about fifty peasants were employed in lading such goods as were admitted to *pratique*, after their period of purification had been accomplished. Here we also found carriages for hire, two of which we immediately engaged to convey some of our party to the celebrated Baths of Mahadia; which, being situated off our road, we were anxious to reach as speedily as possible, in order to enable us to secure our passage on board the Steam Packet, that was to leave Drinkova at daybreak the following morning.

Three of the party accordingly took possession of a Caleche, drawn by a trio of wiry-looking little chestnut ponies, harnessed in the most inartificial way in the world, with bridles, traces, and reins of stout cord; while the others mounted one of the country wagons, filled with hay, and dragged by a couple of wild-looking horses.

Never was there a more sincere exhibition of self-gratulation than that with which we passed the boundary gate of the Quarantine ground; and found ourselves beside the tall stone cross that is erected on its outskirt, as if to claim the thanksgiving of the newly-liberated."

